# Assessing the suitability of medial sural artery perforator flaps in tongue reconstruction – An outcome study

**DOI:** 10.1371/journal.pone.0171570

**Published:** 2017-02-09

**Authors:** Shao-Yu Hung, Charles Yuen Yung Loh, Soo-Ha Kwon, Chia-Hsuan Tsai, Kai-Ping Chang, Huang-Kai Kao

**Affiliations:** 1 Department of Plastic and Reconstructive Surgery; Chang Gung Memorial Hospital & Chang Gung University College of Medicine, Taoyuan, Taiwan; 2 Center for Vascularized Composite Allotransplantation, Chang Gung Memorial Hospital; Graduate Institute of Clinical Medical Sciences, Chang Gung University, Taoyuan, Taiwan; 3 Department of Otolaryngology-Head & Neck Surgery, Chang Gung Memorial Hospital, Chang Gung University College of Medicine, Taoyuan, Taiwan; BG Trauma Center Ludwigshafen, GERMANY

## Abstract

**Introduction:**

Oncological resection of the tongue can be reconstructed using a multitude of free flaps. The medial sural artery perforator (MSAP) flap has been well described in the literature in terms of its anatomy and harvest. However, functional outcome studies of post-reconstruction tongue defects using the MSAP flap have not been reported. This study represents the largest outcome study of patients with tongue reconstructions using MSAP flaps and a comprehensive review of its use.

**Materials and methods:**

From December of 2010 to October of 2015, 579 patients with subtotal glossectomy and free flap reconstructions in Chang Gung Memorial Hospital were retrospectively reviewed. 27 patients were reconstructed with MSAP flap. The pre- and intra-operative factors, as well as flap-related factors were analyzed. Post-operative complications and functional outcomes were evaluated. Donor site assessment were also conducted.

**Results:**

A 96.3% flap survival rate was found with an average total operating time of 6 hours and 18 minutes. 84.6% of patients had primary closure of the donor site with and the remaining either had skin grafts or delayed closure. Donor site closure can be achieved primarily with no functional deficit. Speech intelligibility remained for most patients. 100% of patients resumed normal oral feeding.

**Conclusion:**

The MSAP flap is a small to medium sized flap most suited for subtotal glossectomy defects where optimal outcomes can be achieved in terms of speech clarity and restoration of oral intake.

## Introduction

As the repertoire of free flap options for reconstructing head and neck defects post oncological resection increases, choosing the right flap for reconstructing a particular subunit of the head and neck region may be difficult. The adage ‘one flap for all’ may not be particularly applicable given the ever increasing armamentarium of flaps a reconstructive surgeon may possess. Survival rates for head and neck cancer patients have increased with improvements in surgical technique and oncological management.[1,2] As such, a surge of recurrent tumors which necessitate resection and sequential free flap reconstruction are seen.[3] As such, choosing the most appropriate flap for subunit based reconstruction in head and neck is crucial, and one can reserve more commonly used flaps for recurrent lesions in the future.[4]

Subtotal glossectomies are commonly performed for isolated lesions of the tongue and reconstructing the tongue requires a flap of suitable size and thickness. Much of the literature at present describe and compare the use of fasciocutaneous flaps such as the anterolateral thigh (ALT) flap or the free radial forearm flap in the reconstruction of tongue defects.[5–8] A properly chosen flap for reconstruction of the hemi tongue or subtotal tongue defect will not only ensure that donor site morbidity is decreased with no flap wastage, but also will enhance tongue mobility when the oral space is not fully occupied. The medial sural artery perforator (MSAP) flap, first described by Cavadas et al, is a relatively suitable flap for this purpose.[9] The thin, pliable nature along with its minimal donor site morbidity make this flap ideal for tongue reconstructions where a relatively small volume is needed to be replaced. There is a paucity in the literature at present regarding the use of the MSAP flap in reconstruction of subtotal glossectomy defects and their associated outcomes. In this study, we highlight the outcomes achieved in tongue defect reconstructions using the MSAP flap and review its feasibility as a workhorse flap in tongue reconstructions.

## Materials and methods

This study received Chang Gung Memorial Hospital (CGMH) institutional review board (IRB) permission under study number 201600973B0. Participants had IRB approved written consent to participate in the study. Documentation of consent was recorded in the medical records. The CGMH ethics committee approved this consent procedure. From December of 2010 to October of 2015, 579 patients with tongue cancer who had glossectomies and free flap reconstructions at Chang Gung Memorial Hospital were retrospectively reviewed. 30 patients were identified who received subtotal glossectomies with MSAP flap reconstruction. The inclusion criteria were limited to include only patients where MSAP flaps were only used for subtotal tongue reconstruction and not for other concurrent defects such as the floor mouth.

A total of 30 patients were initially found and 3 cases were excluded. 2 patients had extensive resection margins with segmental mandibulectomies. The MSAP flap in these patients was used only for mouth floor coverage. The other case that was excluded was a patient with a rapidly recurring tumor (within a month) which affected our postoperative evaluation of MSAP flap function. As such, 27 patients were identified that fit our study criteria and were selected for retrospective analysis.

Patient demographics and tumor characteristics were analyzed. Intraoperative details such as flap size, the extent of resection, flap characteristics such as the number of perforators found and pedicle length were obtained. The total operation time was also noted. Donor site closure (primary, delayed or skin grafts) were also documented. Any complications and flap failure were also included in our study. The selected patients were then recalled for donor site evaluation both through clinical evaluation and a patient questionnaire. Any hypertrophic scarring, itching, pigmentation, paraesthesia, perceived social stigma or functional impairment were documented. These were then correlated to the method of donor site closure.

The patients had speech and deglutition evaluated at the time of this study when they were recalled back to the clinic for evaluation. Speech was assessed using the perceptual evaluation described by Taguchi [10] in a 5 point Scale. Deglutition was assessed using a simplified version of the swallowing ability scale described by Fujimoto Y et al. [11] Two independent clinicians evaluated each patient for their speech quality and were given a speech score. Their deglutition was also evaluated and each patient was individually assessed.

## Results

The patients that particularly fit our inclusion criteria all had a background of betel nut consumption, alcohol and tobacco use. All 27 patients had squamous cell carcinoma lesions on the tongues with a stage ranging from T1N0 to T3N2. 16 cases were of primary origin and 11 were recurrent lesions. There were 22 male and 5 female patients in the group collected with an age ranging from 29 to 73 years of age (Table 1).

**Table 1:** Demographics of patients.

	*n* (%)
**Age, years**	
** Mean ± SD**	52.4 ± 11.5
**Gender**	
** M**	23 (85.2)
** F**	4 (14.8)
**BMI**	
** Mean ± SD**	23.3 ± 2.6
**Betel nut**	21 (77.8)
**Smoking**	25 (92.6)
**Tumor stage**	
**T stage**	
**I**	9 (33.3)
**II**	15 (55.6)
**III**	2 (7.4)
**IV**	1 (3.7)
**N stage**	
**0**	20 (74)
**I**	2 (7.4)
**II**	5 (18.5)
**Overall stage**	
**I**	9 (33.3)
**II**	10(37)
**III**	1 (3.7)
**IV**	6 (22.2)
**Tumor status**	
** Primary**	17 (62.9)
** Recurrent**	5 (18.5)
** 2nd primary**	5 (18.5)
**Pre-op R/T**^a^	2
**Pre-op C/T**^b^	2
**Mandibulectomy**	
** Marginal**	6 (22.2)
** Segmental**	0

^a^ R/T, radiotherapy;

^b^ C/T, chemotherapy

All patients were treated with subtotal glossectomies only and were all reconstructed with MSAP flaps. The flap size harvested was from 4 x 9 cm to 5 x 15 cm. The flaps harvested had perforator numbers ranging from 1 to 3 perforators. The average pedicle length of the MSAP flaps harvested for reconstruction of the defects was 12.1 cm. The average operating time was 6 hours and 18 minutes where simultaneous flap harvest and oncological resection was carried out (Table 2). The average flap thickness was 5.2mm in our series which makes this flap a thin and pliable flap that can be folded and contoured to fit a subtotal glossectomy defect.

**Table 2:** Flap details.

	*n* (%)
Resection type	
1/2 of tongue	24 (88.9)
1/2–2/3 of tongue	3 (11.1)
Total	0
Defect size	
Length (cm)	9.3 ± 3.3
Width (cm)	4.8 ± 0.6
Flap size	
Length (cm)	12.1 ± 2.6
Width (cm)	5.2 ± 0.7
Flap type	
Fasciocutaneous	27 (100)
Perforator No.	1.8 ± 0.5
Pedicle length (cm)	12.7 ± 1.3
Flap thickness (mm)	5.2 ± 0.6
Flap harvest time(min)	47 ± 16.3
Total op time (min)	417 ± 86.7
Donor site closure	
Primary	23 (85.1)
Shoelace	3 (11.1)
STSG^a^	1 (3.7)

^a^ STSG, split thickness skin graft

Each patient was evaluated and a speech assessment score was marked by two clinicians. The average of the scores was recorded. With a score of 1 where nothing was understood to a score of 5 where complete comprehension of speech was possible, 13 patients (60%) had a speech score of 5 and 9 patients (40%) had a speech score of 4. The patients were then assigned a deglutition score based where a score of 1 represented tube dependent feeding and 4 a regular oral diet consisting of both solids and liquids. 16 patients had a full score of 4, capable of both solid and liquid diets without problem. 5 patients had a score of 3 points where they could only cope with soft diets at present and had ongoing rehabilitation. This was related to their lack of support at home and their recent recovery from their operation. Lastly, 1 patient could only still manage a liquid diet having just been discharged from hospital 3 weeks prior (Table 3).

**Table 3:** Outcomes and complications.

	*n* (%)
Recipient site complications	
Wound infection	3 (11.1)
Acute complication within one week	
artery occlusion	1 (3.7)
vein occlusion	3 (11.1)
neck hematoma	1 (3.7)
Flap loss	1 (3.7)
Donor site complications	1 (3.7)
Interval between tracheostomy and decannulation (day)	12.6 ± 4.8
Interval between intubation and extubation (day)	1.11 ± 0.3
Speech score (1–5)	
5	14 (60.9)
4	9 (39.1)
≤ 3	0
Deglutition score (0–4)	
4	17 (73.9)
3	5 (21.7)
≤ 2	1 (4.3)

Among them, 23 patients had successful primary closure of the donor site whereas 4 patients had either delayed primary closure or skin grafts. At follow-up outpatient visits, 3 patients had unfortunately passed away. The remaining 23 patients were examined for the following parameters including hypertrophic scarring, itch, change in pigmentation, altered sensation or paraesthesia and perceived social or functional impairment with regards to their donor site scars. 9 of the remaining 19 patients with primary closure of the donor site had reported itching of the scar and had evidence of hypertrophic scarring and hyperpigmentation. Patients who had skin grafts all reported positive for at least one of the parameters stated above. The single patient who had delayed primary closure of his donor site reported hypertrophic scarring associated with hyperpigmentation and itching. None of the patients reported any subjective social stigma or functional impairment related to their donor site (Table 4).

**Table 4:** Assessment of donor site scar.

	Total (*n* = 24)	Primary closure (*n* = 20)	Skin graft (*n* = 3)	Shoelace (*n* = 1)
Hypertrophic scar	5	3/20	1/3	1/1
Itching	6	4/20	1/3	1/1
Pigmentation	5	2/20	2/3	1/1
Numbness of skin graft	0	0	0	0
Paresthesia of skin graft	1	0	1/3	0
Social stigma	0	0	0	0
Functional impairment (subjective)	0	0	0	0

## Discussion

**Fig 1:**
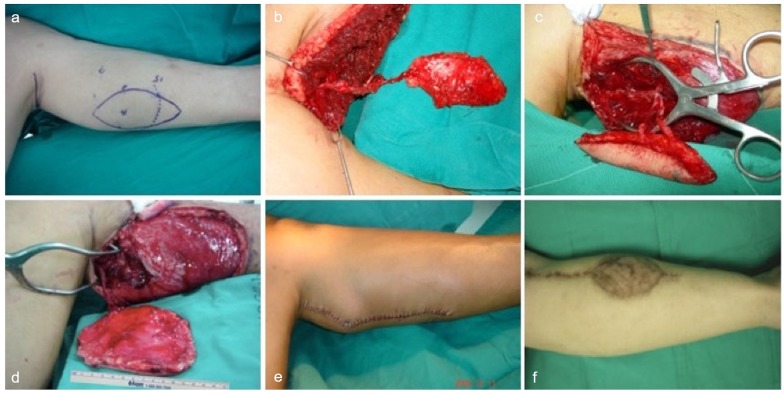
(a) Preoperative marking of the MSAP flap with the inferior border of media gastrocnemius muscle and ultrasound Doppler marking of the perforator. (b) Intraoperative MSAP flap harvest with one perforator and its pedicle. (c) Intraoperative MSAP flap demonstrating a two perforator harvest. (d) Intraoperative photograph demonstrating the extent of MSAP flap size harvested. (e) Primary closure of the donor site. (f) Photograph demonstrating skin graft closure of the donor site.

**Fig 2:**
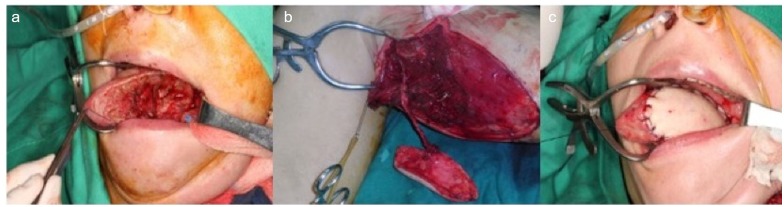
(a) Intraoperative photograph demonstrating a subtotal hemiglossectomy defect. (b) Intraoperative harvest of a large MSAP flap from an overweight patient. The MSAP flap was of a suitable size for reconstruction of a subtotal glossectomy defect. (c) Photograph demonstrating the inset of the MSAP flap with good contouring and restoration of tongue form.

Microsurgical free tissue transfer for head and neck reconstruction in cancer patients has become the first rung on the reconstructive ladder which achieves both functional and aesthetic restoration. A successful tongue reconstruction should restore swallowing and speech function as well as cosmesis. The concept and design of a customized flap is to choose an ideal flap to fit the 3-D arrangement of a defect in a one-stage reconstruction with minimal donor site morbidity.[12] For tongue reconstruction, the speech intelligibility is closely dependent on the mobilization of the remaining native tongue, and the deglutition function is strongly related to the volume of the reconstructed tongue. An ideal flap for tongue reconstruction has to provide both tissue bulk and mobilization. The MSAP flap has been demonstrated to be a versatile flap in head and neck reconstruction associated with minimal donor site morbidity.[13] The volume of the flap with a reported average area of 65 cm^2^ provides optimal volume and pliability for the reconstruction of a subtotal glossectomy defect as shown in our study.[14] (Fig 1) The adequate pedicle length allows the flap to reach commonly used recipient vessels in the neck such as the superior thyroid artery and the facial artery if required. Tongue reconstructions in general can be performed using any fasciocutaneous flap. However, certain considerations such as size and volume, donor site morbidity, ease of harvest and a strategized use of flaps where defects are sized appropriately to the amount of tissue provided by a particular flap.[15] In patients with subtotal glossectomy defects, the use of the MSAP flap provides comparable volume replacement with pliability and suppleness required for adequate tongue mobility without obliterating the oral cavity. (Fig 2) The donor site of the MSAP flap at the lower extremity of the patient allows for adequate space between the team performing the oncological resection and the reconstructive surgeon.

The learning curve of the MSAP flap is definitely longer than more conventional fasciocutaneous flaps due to the varied location of the perforator and long intramuscular dissection which can prove tedious to the unfamiliar. However, the preoperative use of Doppler perforator mapping is routinely performed at our institution and can help with locating a sizable perforator. Preoperative computed tomography angiography (CTA), although not routinely used in our institution, may be used to evaluate the vascular condition of the MSAP flap especially in patients with a history of diabetes mellitus or previous trauma. Other preoperative modalities such as magnetic resonance angiography or color flow Doppler may add additional valuable information with regards to recipient vessel status and patency.[16] An endoscopic approach allows for close-up visualization of perforators which can help with flap design around the perforator. A direct approach using a 4cm incision laterally may be used to visualize the perforator before incorporating the incision into the flap design once the perforator is determined to be usable. Our landmarks used in the harvest of MSAP flaps result in four lines that help guide the location of perforators. A line across the popliteal crease is first drawn, followed by the midpoint of the popliteal crease to the Achilles tendon, then a third line along the medial border of the gastrocnemius and lastly a line drawn from the medial tibial condyle to the center of the medial malleolus. The main perforator of the medial sural artery can be then located using a hand held Doppler device 6cm along the line from the popliteal crease to the distal border of the medial gastrocnemius muscle. The patient is placed in semi frog like position where flexion of the knee and the hip is abducted. This allows for adequate exposure of the intended surgical site. Placing the patient in stirrups or shortening of the operating table may be performed to allow the operator to sit in closer proximity to the operating field, hence improving intraoperative ergonomics for the surgeon. The MSAP flap may be harvested with or without the use of a tourniquet. Tourniquet control allows for a bloodless operating field whereas without a tourniquet, the operator may better observe the pulsation of the perforator, aiding its assessment.

**Fig 3:**
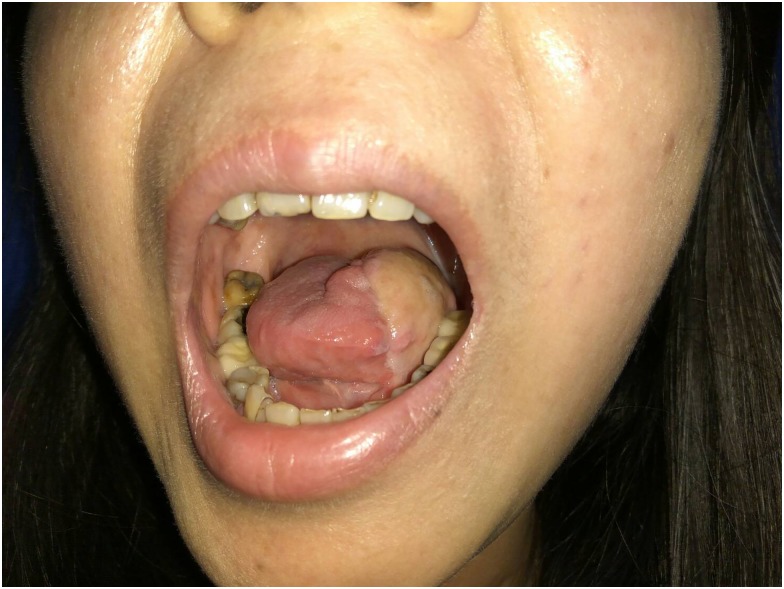
Postoperative photograph of a hemiglossectomy defect reconstructed with a MSAP flap.

The functional outcome of tongue reconstructions using MSAP flaps can be optimal as shown in our study with a relatively quick return to oral intake and good speech quality restoration. Tongue resection, especially after subtotal glossectomies, requires a relatively smaller flap that can be easily mobilized by the remaining tongue muscles, but also be large and firm enough to allow complete adherence to the hard palate during phonation. This balance in size and volume is crucial in obtaining a good functional result in terms of tongue mobility for swallowing and sealing off spaces in the oral cavity during phonation for good speech. (Fig 3) ALT flaps and free radial forearm flaps also provide optimal and desirable functional outcomes in tongue reconstructions. The major difference between the three flaps, however, is the donor site morbidity in our opinion when considered in conjunction with the defect size and indication.

A good outcome in terms of donor site morbidity is seen in our study with scar quality complaints being the commonest complication of the flap. This could be attributed to the patient population characteristics of our study and designing the flap with primary closure of the donor site being the best method of minimizing poor scarring. Skin grafts and delayed wound closure methods, as known from their increased inflammation and poor quality of scar formation, were indeed associated with a greater rate of poor quality scarring and possibly altered sensation as well. ALT flaps also result in a relatively concealed scar but sacrificing the lateral cutaneous nerve of the thigh during harvest may result in discomfort to the patient.[17] The use of an ALT for a small defect in our cases would be an underutilization of the ALT flap where it could be used to reconstruct larger resections in the future if the tumor should recur. Free radial forearm flaps have an unsightly scar at the donor site where skin grafts are most likely required for the flap volume required for a subtotal glossectomy reconstruction. The disruption of the conjoint tendon during harvest may also result in unsightly bowstringing of wrist flexors which may not be favorable in certain patients. Lastly, if disruption to the paratenon covering the tendons during harvest occurs, poor skin graft take may result and may require subsequent revision surgery.[18]

The main limitation of the study was the subtotal glossectomy nature of the patients where one could arguably state that functional deficit was not as large compared to a total glossectomy. However, the MSAP flap is only suitable for small sized defects in which subtotal glossectomies fit its use. MSAP flaps are not clinically indicated in total glossectomies and hence we were not able to assess the function of this group of patients in this study. The observational and retrospective nature of the study limited the level of evidence assessed but instead provides a cross-sectional study of the characteristics of the patients suitable for MSAP flap reconstruction and their associated outcomes.

## Conclusion

The MSAP flap has been shown to work well for subtotal glossectomy reconstructions in our series of patients where volume to be reconstructed only involves the subtotal glossectomy volume and not for coverage of the mouth floor. An acceptable outcome and restoration can be expected with the use of such flaps for subtotal glossectomy reconstruction.
